# MicroNet-MIMRF: a microbial network inference approach based on mutual information and Markov random fields

**DOI:** 10.1093/bioadv/vbae167

**Published:** 2024-10-28

**Authors:** Chenqionglu Feng, Huiqun Jia, Hui Wang, Jiaojiao Wang, Mengxuan Lin, Xiaoyan Hu, Chenjing Yu, Hongbin Song, Ligui Wang

**Affiliations:** Department of Epidemiology and Health Statistics, School of Public Health, China Medical University, Shenyang 110122, China; Department of Infectious Disease Prevention and Control, Chinese PLA Center for Disease Control and Prevention, Beijing 100071, China; Department of Infectious Disease Prevention and Control, Chinese PLA Center for Disease Control and Prevention, Beijing 100071, China; Department of Infectious Disease Prevention and Control, Chinese PLA Center for Disease Control and Prevention, Beijing 100071, China; The State Key Laboratory of Multimodal Artificial Intelligence Systems, Institute of Automation Chinese Academy of Sciences, Beijing 100190, China; The Academy of Military Medical Sciences, Academy of Military Science of Chinese People’s Liberation Army, Beijing 100071, China; Department of Infectious Disease Prevention and Control, Chinese PLA Center for Disease Control and Prevention, Beijing 100071, China; Department of Infectious Disease Prevention and Control, Chinese PLA Center for Disease Control and Prevention, Beijing 100071, China; Department of Infectious Disease Prevention and Control, Chinese PLA Center for Disease Control and Prevention, Beijing 100071, China; Department of Infectious Disease Prevention and Control, Chinese PLA Center for Disease Control and Prevention, Beijing 100071, China

## Abstract

**Motivation:**

The human microbiome, comprises complex associations and communication networks among microbial communities, which are crucial for maintaining health. The construction of microbial networks is vital for elucidating these associations. However, existing microbial networks inference methods cannot solve the issues of zero-inflation and non-linear associations. Therefore, necessitating novel methods to improve the accuracy of microbial networks inference.

**Results:**

In this study, we introduce the Microbial Network based on Mutual Information and Markov Random Fields (MicroNet-MIMRF) as a novel approach for inferring microbial networks. Abundance data of microbes are modeled through the zero-inflated Poisson distribution, and the discrete matrix is estimated for further calculation. Markov random fields based on mutual information are used to construct accurate microbial networks. MicroNet-MIMRF excels at estimating pairwise associations between microbes, effectively addressing zero-inflation and non-linear associations in microbial abundance data. It outperforms commonly used techniques in simulation experiments, achieving area under the curve values exceeding 0.75 for all parameters. A case study on inflammatory bowel disease data further demonstrates the method’s ability to identify insightful associations. Conclusively, MicroNet-MIMRF is a powerful tool for microbial network inference that handles the biases caused by zero-inflation and overestimation of associations.

**Availability and implementation:**

The MicroNet-MIMRF is provided at https://github.com/Fionabiostats/MicroNet-MIMRF.

## Graphical abstract

**Graphical abstract:**
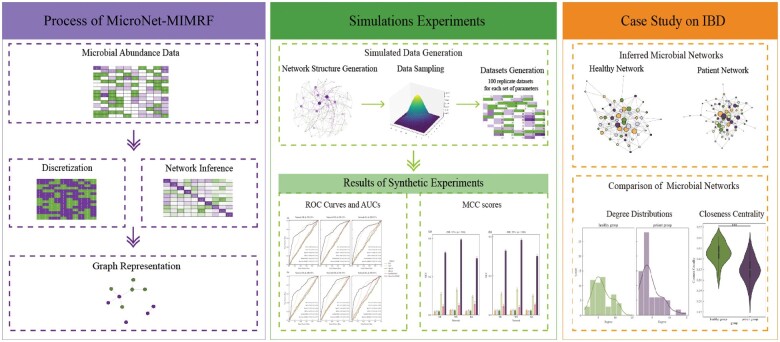

## 1 Introduction

The human microbiome, often referred to as the secondary genome, plays a critical role in maintaining human health (Dominguez-Bello *et al.* 2019, Aggarwal *et al.* 2023). The microbial organisms within this ecosystem have rarely been isolated, instead forming cohesive communities characterized by intricate associations (Dundore-Arias *et al.* 2023). Advances in high-throughput sequencing techniques, such as 16S rRNA sequencing and metagenome shotgun sequencing, have enabled researchers to directly analyze microbial DNA from environmental samples without the need for individual cultivation in laboratories (Ha *et al.* 2020, Jiang *et al.* 2020). Consequently, microbial abundance profiles can be obtained; these profiles can provide information about indirect interactions and associations among microbes (Yang *et al.* 2017). Such associations form complex microbial networks or microbial co-occurrence networks that can be characterized using network inference tools to elucidate the underlying structures and patterns of microbial communities.

A critical step in constructing microbial networks is to infer pairwise associations between taxa. These associations can be divided into two categories: linear associations (e.g. the relationship between single-species populations and antibiotic concentration) and non-linear associations (e.g. the interactions between different microbial communities) (Beardmore *et al.* 2018). Traditional methods for estimating these associations include correlation-based methods (Pinto *et al.* 2022, Li *et al.* 2023), molecular ecological network analysis (MENA) (Deng *et al.* 2012), and sparse correlations for compositional data (SparCC) (Friedman and Alm 2012). Correlation-based methods that use Pearson’s correlation coefficient (PCC) or Spearman’s rank correlation coefficient (SCC) are straightforward and computationally efficient and effectively capture linear correlations between microbial taxa. MENA, which utilizes random matrix theory to establish thresholds, has been widely applied in diverse fields, such as the gut (Hong *et al.* 2024), environment (Guo *et al.* 2022), and plant microbiomes (Hu *et al.* 2021). MENA is based on SCC to infer correlations, and it effectively reduces false positive correlations by establishing noisy thresholds using random matrix theory. SCC and MENA are based on the ranks of data to calculate the correlation coefficient and can capture monotonic non-linear relationships between two variables. Despite their utility, these correlation methods fail to detect non-monotonic, non-linear relationships and could not address the compositional bias inherent in relative microbial abundance data. This bias can lead to an overestimation of microbial associations (Liu *et al.* 2021). SparCC addresses compositional bias by applying a log-ratio transformation to relative microbial abundance data and using inverse covariance to estimate associations (Kurtz *et al.*, 2015). However, these three methods overlook the zero-inflation feature in microbiome data, in which excessive zeros can result from both the true absence and insufficient sequencing depths.

Mutual information, which is derived from information theory, measures non-linear relationships between variables and has been applied to construct various biological networks, including protein-protein interaction networks (Arcos *et al.* 2023), weighted gene co-expression networks (Rau *et al.* 2013), and microbial networks (Mokhtari and Ridenhour 2022). Although mutual information can capture the associations between taxa with low abundance, direct applications may overestimate these interactions (Ullmann *et al.* 2023). In contrast, Markov random fields (MRFs) measure conditional dependencies between variables, distinguish direct from indirect interactions, and enhance network interpretability (Cai *et al.* 2019).

Microbial abundance profiles obtained from high-throughput sequencing exhibit high sparsity and zero-inflation (Jiang *et al.* 2023). COZINE infers sparse microbial networks based on the multivariate Hurdle model (Ha *et al.* 2020), while HARMONIES utilizes the zero-inflated negative binomial model along with Gaussian graphical models to process microbial abundance data and infer microbial networks (Jiang *et al.* 2020). These approaches apply different zero-inflation models to address the issue of zero-inflation in microbiome data. COZINE and HARMONIES take advantage of sparse inference to reduce the complexity of microbial networks, emphasizing only the most important microbial associations. Nevertheless, a comprehensive comparison of microbial networks comprising diverse microbial communities offers researchers a broader perspective. Thus, we believe that constructing microbial networks that are not overly sparse also holds significant importance. Moreover, both COZINE and HARMONIES require a substantial sample size, a requirement that many actual microbiome studies are hard to fulfill. Developing a method a method applicable to a wide range of sample sizes would be of considerable practical value. Microbial abundance data, which are essentially count data, are often modeled using the Poisson distribution. However, a zero-inflated Poisson (ZIP) distribution provides a more accurate representation, because of zero-inflation (Xu *et al.* 2021).

To address the issues of non-monotonic non-linear associations and zero-inflation, we propose the Microbial Network based on Mutual Information and Markov Random Fields (MicroNet-MIMRF). This novel approach involves the following steps: (i) Discretization: Converting abundance vector *X* into a binary vector *Y* based on the ZIP model expectation; (ii) Network inference: Applying an MRF based on mutual information with a simulated annealing algorithm to estimate pairwise conditional dependencies between binary vectors; (iii) Graph representation: Representing these dependencies as an undirected and unweighted graph, denoted as *G* = (*V*, *E*), where the taxa are nodes and the associations are edges.

The effectiveness of MicroNet-MIMRF is validated through simulations, which demonstrate that MicroNet-MIMRF outperforms other common methods. Additionally, we apply MicroNet-MIMRF to data from the Inflammatory Bowel Disease Multi'omics Database (http://ibdmdb.org) to illustrate the unique associations detailed in this study.

## 2 Methods

### 2.1 Methods overview

**Figure 1.**
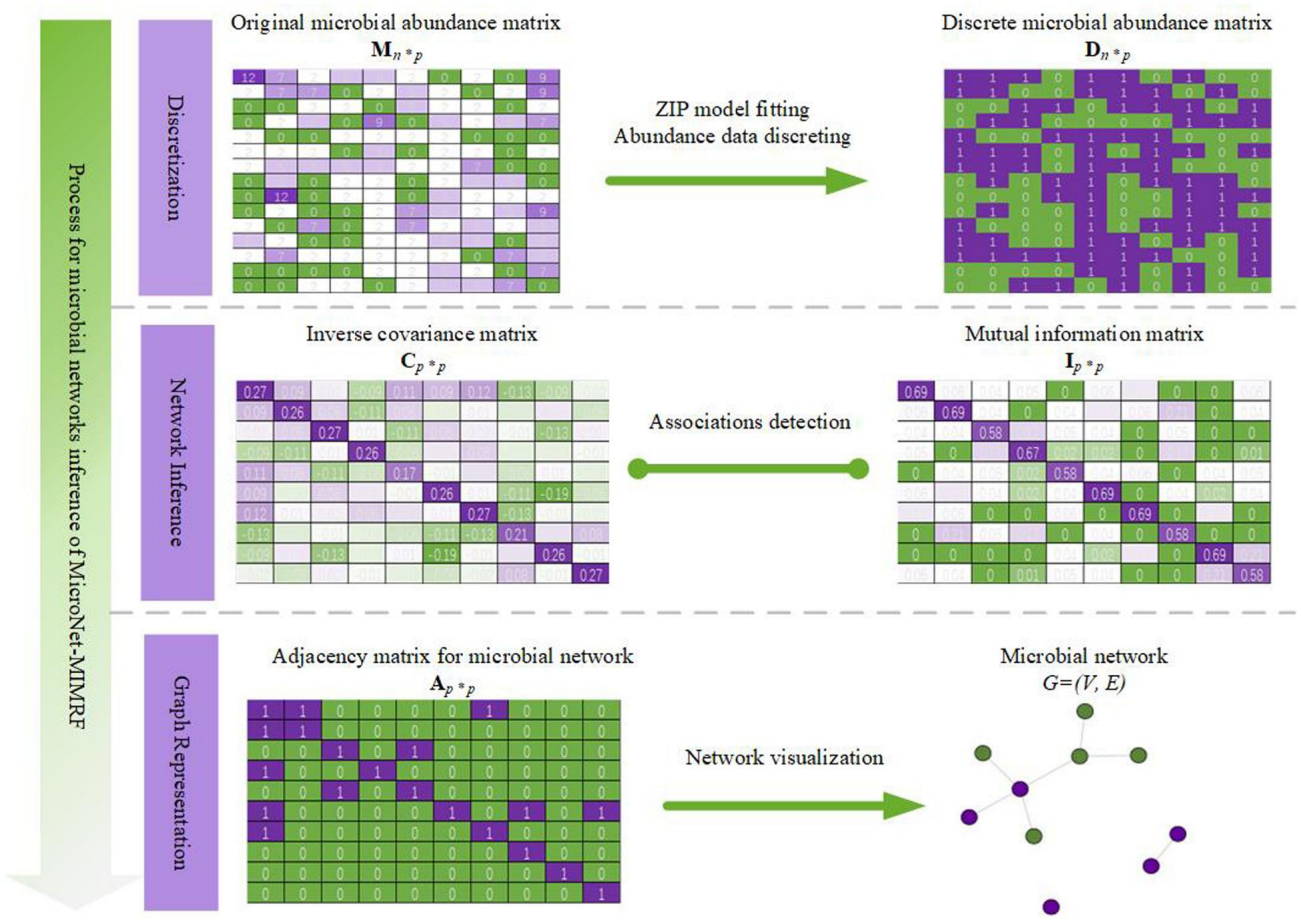
Schematic diagram of the MicroNet-MIMRF framework.

Figure 1 illustrates the process for microbial networks inference of MicroNet-MIMRF. Assuming a microbial abundance matrix, **M**_*n×p*_, where *n* represents the number of samples and *p* represents the number of taxa, we can easily obtain this matrix using 16 s rRNA sequencing or metagenomic shotgun sequencing. Each element *x_ij_* (*x_ij_* ∈ **M**, *i *=* *1,2,…,*n*, *j *=* *1,2,…*p*) indicates the read counts of taxon *j* in sample *i*. Given that microbial abundance data are count data characterized by zero-inflation, we used the ZIP model to model *x_ij_*:

(1) $$x_{ij}:\{\begin{array}{c} \phi_{i}+\left(1-\phi_{i}\right)e^{-\lambda_{ij}},\;\;x_{ij}=0\\ \left(1-\phi_{i}\right)\frac{\lambda_{ij}^{x_{ij}}}{x_{ij}},\;\;\;\;\;\;\;x_{ij}\geq 1. \end{array}$$

Here, *ϕ_i_* is the probability mass accounting for inflated zeros, which can be interpreted as extra zeros due to insufficient sequencing depth and coverage. *λ_ij_* represents the mean abundance of taxon *j* in sample *i*. The expectation of this distribution can be measured using *E*(*x_ij_*):

(2) $$E\left(x_{ij}\right)=\left(1-\phi_{i}\right)\lambda_{ij}.$$

To address the challenges arising from inflated zeros and compositional bias, we propose discretizing the matrix **M**_*n×p*_ into a discrete microbial existence matrix **D**_*n×p*_ based on a prospective abundance threshold derived from the expectation of the ZIP model. In this discretized matrix, a value of one signifies the presence of a taxon, whereas a value of zero indicates its absence or abundance below the detection limit. The discretization process can be divided into two steps: (i) Compute expectation: Calculate the expectation *E*(*x_ij_*) for each *x_ij_* using (2). (ii) Set threshold: The expectation is used as the threshold. If *x_ij_* is greater than or equal to its expectation, it is marked as one; otherwise, it is marked as zero. This helps to distinguish true rare taxa from false negatives due to technical limitations.

After obtaining the discrete matrix **D**_*n×p*_, the following steps are performed to construct the microbial network:

1. Calculate the inverse covariance matrix. First, we calculate the inverse covariance matrix **C**_*p×p*_, where each element *c_jk_* (*c_jk_* ∈ **C**, *j* = 1,2,…,*n*, *k* = 1,2,…*p*) indicates associations or conditional independence relationships between taxon *j* and taxon *k*. Specifically, positive *c_jk_* and negative *c_jk_* indicate different directions of the association relationship. We used the absolute value of *c_jk_*, denoted as |*c_jk_*|, for microbial network inference, because we aimed to construct an undirected network.
2. Apply a binary MRF based on mutual information. Simultaneously, we employ a binary MRF based on mutual information to capture conditional dependencies or non-linear associations between two taxa. Mutual information is a useful measure for detecting correlations between two variables and is widely used in bioinformatics analysis. Given the variables *J* and *K*, their mutual information *I*(*J*; *K*) is computed as follows:
  (3) I(J;K)=H(J)−H(J|K),
  where the variables *J* and *K* are the binary abundance vectors of taxon *j* (*j* = 1,2,…*p*) and taxon *k* (*k *=* *1,2,…*p*) in the matrix **D**_*n×p*_ across all samples. Each binary abundance vector, such as vector **j**, is one dimensional with *n* elements.
3. Information entropy calculation. Information entropy *H*(*J*) is the average amount of abundance information for taxon *j*, formulated as
  (4) $$H\left(J\right)=\sum_{j\in J}p\left(j\right)\;\text{log}\;\frac{1}{p\left(j\right)}.$$
  The conditional entropy *H*(*J|K*) is defined as
  (5) $$H\left(J|K=k\right)=\sum_{j\in J}p\left(j|k\right)\;\text{log}\;\frac{1}{p\left(j|k\right)}.$$
4. Optimizing for maximum mutual information. For each taxon *j* (*j* = 1,2,…*p*), *p−*1 elements of *I*(*J*; *K*) exist (where *j* ≠ *k*), forming a vector **i** that consists of these *p−*1 elements. In the MRF, we aim to optimize the graph structure to maximize the total mutual information of connected pairwise taxa, using a simulated annealing algorithm as the iterative optimization method. This results in the Maximum Mutual Information Association algorithm (MMIA), which calculates the optimized mutual information matrix **I**_*p×p*_.
5. Constructing the network adjacency matrix. Based on the intersection of the inverse covariance matrix **C**_*p×p*_ and mutual information matrix **I**_*p×p*_, we transform **I**_*p×p*_ into the network adjacency matrix **A**_*p×p*_. This matrix reflects the pairwise associations between two taxa, where one indicates that two taxa are associated, and zero indicates that the taxa are non-associated. The sign (positive or negative) of the inverse covariance matrix **C**_*p×p*_ is used to reveal the sign of pairwise associations.
6. Construction of the microbial network. By following the steps mentioned above, we can construct the microbial network, denoted as *G* = (*V*, *E*).

### 2.2 MMIA algorithm

The MMIA algorithm in Algorithm 1 (Table 1), inspired by the mLDM model (Yang *et al.* 2017), aims to identify significant pairwise associations among taxa. The core of this algorithm is the use of MRFs for structural modeling of the microbial network, which helps reduce redundant associations and enhances the interpretability of the microbial network. A microbial network is an undirected graph represented as *G* = (*V*, *E*), where *V* represents the taxa of microbes and *E* represents the interactions or pairwise associations between microbes. In this graph, the states of the microbes are interdependent and adhere to local Markov properties. Thus, the state of one microorganism is conditionally independent of the states of the other microorganisms, given their neighbors. Therefore, the MRF effectively models and captures the dependencies between microorganisms.

**Table 1.** The pseudo-code for MMIA algorithm.

**Algorithm 1:** MMIA algorithm.
**Input:** discrete microbial abundance matrix **D**, maximum iteration count S, initial temperature T, cooling rate α **Output:** optimized mutual information matrix **I** **Procedure:** 1. Initialize: set the initial temperature T, set the maximum iteration count S 2. Simulated annealing loop: While T > 0 do For *a *=* *1 to S do For j = 1 to *p* do 1) Calculate MMI(initial) = max(**I**^’^) using Equation (3) 2) Generate a new MRF denoted as D* 3) Calculate new MMI(new) = max(**I**^’’^) using Equation (3) 4) Acceptance criteria: If MMI(new) > MMI(initial) then Set the best MRF to D* Else Calculate *d* = MMI(initial)-MMI(new) Calculate *p *=* e^-d/T^* Generate a random value *q* between 0 and 1 If *p *>* q* then Set the best MRF to D* End if End if End for End for Cool down: Update temperature: T = α*T Update iteration count: S = max(5, α*S) End while 3. Final calculation: 1) Return final MRF D* 2) Calculate the optimized mutual information matrix **I** using D* in Equation (3) 3) Return the optimized mutual information matrix **I** **End Procedure**

### 2.3 Simulated data generation

To validate the effectiveness of the MicroNet-MIMRF framework, we design several experiments using simulated datasets. We then compare the performance of MicroNet-MIMRF with that of other state-of-the-art methods, including PCC, SCC, SparCC, and MENA. Referring to the “Normal to Anything” principle (Kurtz *et al.* 2015) and the method of mLDM (Yang *et al.* 2017), our simulation datasets are generated as follows: (i) Network structure generation. We generate three different types of networks [Erdős-Rényi random network (ER network), Watts-Strogatz small-world network (WS network), and Barabási-Albert scale-free network (BA network)] to test the ability of MicroNet-MIMRF to recover various network structures. (ii) Precision and covariance matrix generation. First, we generate the precision matrix Ω: For each network structure, we randomly sample values from a uniform distribution of [−0.1, 0.1] to create the precision matrix Ω. Second, we compute the covariance matrix Σ as the inverse of the precision matrix, Σ = Ω^−1^. (iii) Decompose the covariance matrix Σ. Using Cholesky decomposition, we decompose Σ into a lower triangular matrix **L**. The decomposition is Σ = **LL^*T*^**. (iv) Data generation. We generate random variables with the structure of Σ using a multivariate Gaussian distribution, then resample the abundance values for each random variable using a Poisson distribution. (v) Zero-inflation simulation. We emulate the zero-inflation phenomenon commonly observed in microbiome datasets by randomly assigning a certain proportion of counts to zero, with this assignment weighted by abundance. (vi) Parameter selection and replicate dataset generation. For ER and WS networks, we set the connection probability *ρ *= 0.1. For the BA network, we set the number of edges to attach a new node to exiting nodes as *m *=* *2. Then, we define the number of taxa as *p *=* *50 and determine the number of samples as *n *=* *50, 100, or 500. Finally, we establish the zero-inflation rate (ZIR) at 35% and 50%. For each set of parameters, we generate 100 replicate datasets to facilitate a comprehensive evaluation.

### 2.4 Metrics of comparison

To evaluate and benchmark the performance of the different methods on simulated synthetic datasets, we utilize several metrics, including the receiver operating characteristic (ROC) curves, the area under the curve (AUC), and Matthew’s correlation coefficient (MCC). The MicroNet-MIMRF and MENA methods estimate conditional dependencies; therefore, we compare the network structures with those of the true network. The PCC, SCC, and SparCC methods estimate the linear correlation relationships; therefore, we compare the results with those of the true correlation matrix. To obtain the ROC curves and AUC, we plot the true positive rate against the false positive rate across a range of cutoff values. We use the MCC to assess the overall accuracy of the results inferred using the different approaches. The formulas for true positive rate, false positive rate, and MCC are as follows:

(6) $$\mathrm{TPR}=\frac{\mathrm{TP}}{\mathrm{TP}+\mathrm{FN}},$$

(7) $$\mathrm{FPR}=\frac{\mathrm{FP}}{\mathrm{TN}+\mathrm{FP}},$$

(8) $$\mathrm{MCC}=\frac{\mathrm{TP}\times \mathrm{TN}-\mathrm{FP}\times \mathrm{FN}}{\sqrt{\left(\mathrm{TP}+\mathrm{FP}\right)\left(\mathrm{TP}+\mathrm{FN}\right)\left(\mathrm{FP}+\mathrm{TN}\right)\left(\mathrm{FN}+\mathrm{TN}\right)}}.$$

In (6–8), TP and FP represent the numbers of true and false edges in the microbial network, respectively, and TN and FN represent the numbers of true and false non-associations, respectively. The AUC provides a single scalar value to summarize model performance. The AUC ranges from 0 to 1, with values closer to one indicating better performance. MCC assesses the overall accuracy of the results inferred using different approaches and ranges from −1 to 1, with values closer to one indicating higher prediction quality. The ROC curves, AUC, and MCC comprehensively indicate the performance of different methods for recovering the true network structure or correlation matrix from synthetic datasets, enabling us to compare the accuracy and quality of the inferred networks.

## 3 Results

### 3.1 Simulation results

**Figure 2.**
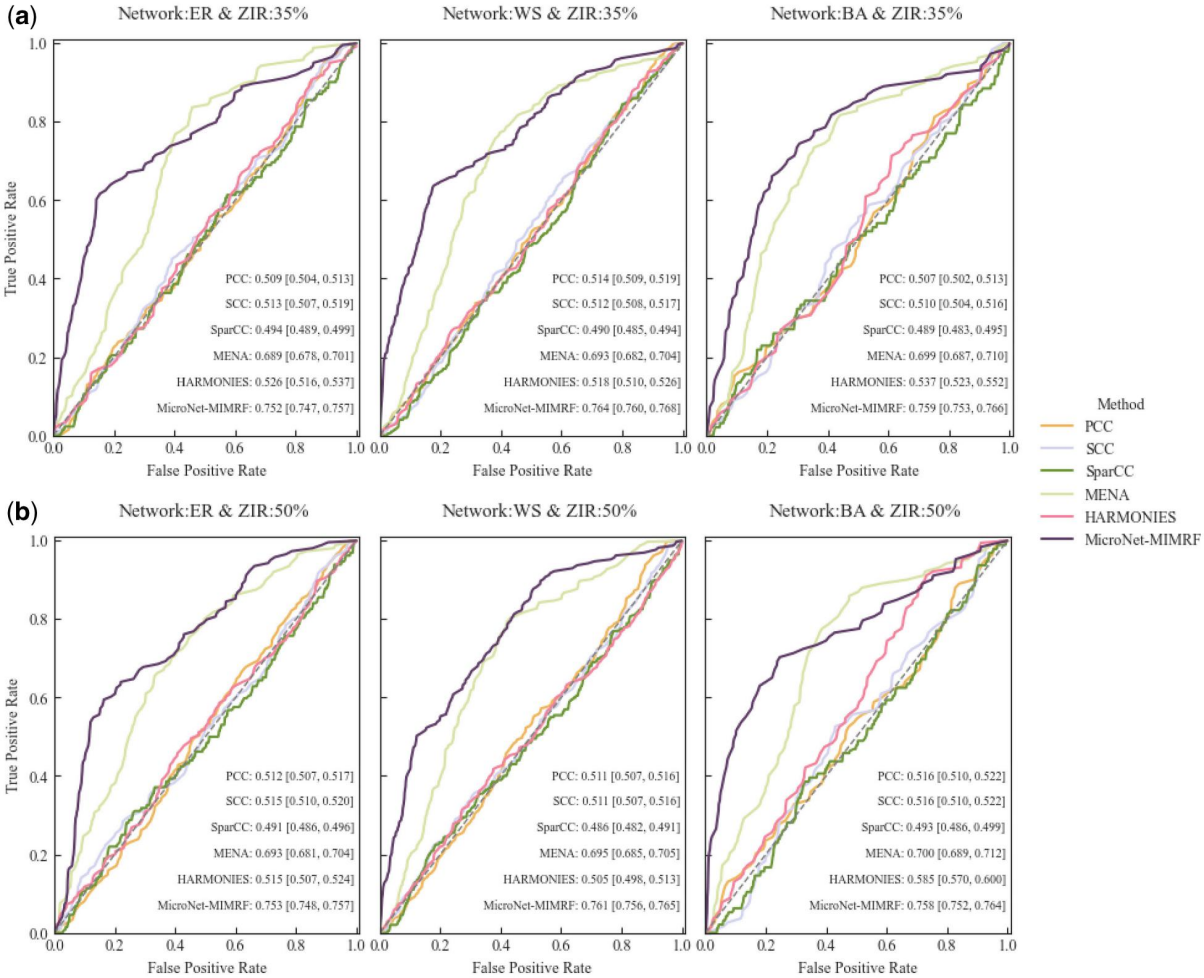
Receiver operating characteristic curves of all methods in the simulation datasets. (a) ZIR = 35%; (b) ZIR = 50%. For each simulation experiment, the number of taxa is *p *=* *50, and the sample size is *n *=* *500. Graphs show the AUC scores among 100 replicated simulations of each method with the same parameters.

To evaluate the performance of the MicroNet-MIMRF method, several comparative experiments were conducted using four existing methods: PCC, SCC, SparCC, MENA, and HARMONIES. Supplementary Figures S1 and S2 and Fig. 2a and b illustrate the ROC curves and AUCs for the microbial network across the three different network structures with simulation parameters of *p *=* *50, *n *=* *50, 100, 500, and ZIR = 35% or 50%. MicroNet-MIMRF demonstrated superior AUC performance across all tested scenarios, particularly in the BA networks. For ZIR = 35%, the AUC of MicroNet-MIMRF reached 0.752 (95% CI 0.747-0.757), 0.764 (95% CI 0.760-0.768), and 0.759 (95% CI 0.753-0.766) across the different network structures. For ZIR = 50%, the AUC of MicroNet-MIMRF were 0.753 (95% CI 0.748-0.757), 0.761 (95% CI 0.756-0.765), and 0.758 (95% CI 0.752-0.764). The methods based on the correlation relationships, such as PCC, SCC, and SparCC, showed comparable AUC performance. However, their AUC values were consistently outperformed by non-linear association-based methods such as MicroNet-MIMRF and MENA. HARMONIES estimated a sparse microbial network, showing low AUCs under different parameters. The simulation data, which are rich in non-linear associations, highlight the capacity of MicroNet-MIMRF to identify and capture complex relationships within microbial networks. Additionally, under small-scale sample size (*n *=* *50) (Supplementary Fig. S1), middle-scale sample size (*n *=* *100) (Supplementary Fig. S2), and large-scale sample size (*n *=* *500) (Fig. 2), MicroNet-MIMRF showed superior AUC performance, indicating its applicability to both small-scale and large-scale sample sizes.

The average AUC scores among 100 replicated simulations are summarized in Table 2. Irrespective of whether ZIR was set at 35% or 50%, MicroNet-MIMRF consistently achieved AUC values over 0.75. This indicates that the MicroNet-MIMRF effectively addresses the issue of zero-inflation and is proficient at constructing microbial networks, even with zero-inflated abundance data. Figure 2a illustrates that MENA and MicroNet-MIMRF showed similar excellent performance for ER networks at both ZIR levels and achieved good performance for WS networks when ZIR was 35%. However, the average AUC scores of MENA were significantly lower than those of MicroNet-MIMRF (*t *=* *26.566, 35.890, 48.627, 32.352, 46.070, and 53.603; all *P *<* *.001), and the AUC standard deviations of MENA were larger than those of MicroNet-MIMRF, indicating the excellent performance and robustness of MicroNet-MIMRF. For other parameter combinations, MicroNet-MIMRF significantly outperformed MENA in terms of AUC values.

**Table 2.** AUC scores of each method with 95% confidence intervals of 100 replicated simulations (*p *=* *50, *n *=* *500).

Network	Method	ZIR: 35%	ZIR: 50%
		AUC (95% CI)	AUC (95% CI)
ER	PCC	0.509 (0.504, 0.513)	0.512 (0.507, 0.517)
	SCC	0.513 (0.507, 0.519)	0.515 (0.510, 0.520)
	SparCC	0.494 (0.489, 0.499)	0.491 (0.486, 0.496)
	MENA	0.689 (0.678, 0.701)	0.693 (0.681, 0.704)
	HARMONIES	0.526 (0.516, 0.537)	0.515 (0.507, 0.524)
	MicroNet-MIMRF	0.752 (0.747, 0.757)	0.753 (0.748, 0.757)
WS	PCC	0.514 (0.509, 0.519)	0.511 (0.507, 0.516)
	SCC	0.512 (0.508, 0.517)	0.511 (0.507, 0.516)
	SparCC	0.490 (0.485, 0.494)	0.486 (0.482, 0.491)
	MENA	0.693 (0.682, 0.704)	0.695 (0.685, 0.705)
	HARMONIES	0.518 (0.510, 0.526)	0.505 (0.498, 0.513)
	MicroNet-MIMRF	0.764 (0.760, 0.768)	0.761 (0.756, 0.765)
BA	PCC	0.507 (0.502, 0.513)	0.516 (0.510, 0.522)
	SCC	0.510 (0.504, 0.516)	0.516 (0.510, 0.522)
	SparCC	0.489 (0.483, 0.495)	0.493 (0.486, 0.499)
	MENA	0.699 (0.687, 0.710)	0.700 (0.689, 0.712)
	HARMONIES	0.537 (0.523, 0.552)	0.585 (0.570, 0.600)
	MicroNet-MIMRF	0.759 (0.753, 0.766)	0.758 (0.752, 0.764)

**Figure 3.**
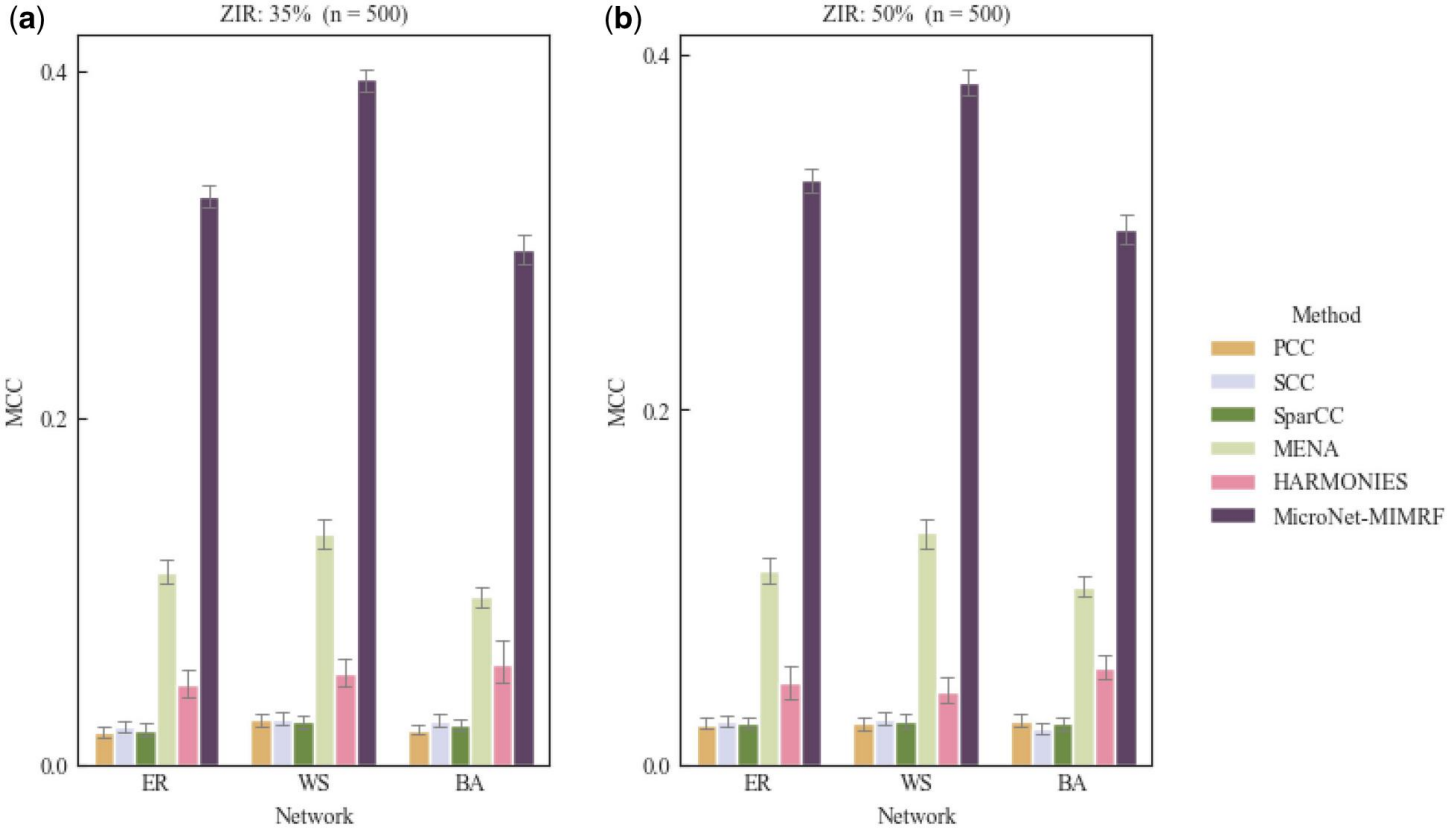
Matthew’s correlation coefficient bar plots of simulation datasets for different methods. (a) ZIR = 35%; (b) ZIR = 50%. For each simulation experiment, the number of taxa is *p *=* *50, and the sample size is *n *=* *500. Graphs show the average results of 100 replicate simulations with the same parameters.

Supplementary Figures S3 and S4 and Fig. 3a and b show that MicroNet-MIMRF achieved the highest MCC scores across the three distinct network structures at varying ZIRs. These results underscore the robustness of MicroNet-MIMRF for inferring microbial networks by accurately estimating the conditional dependencies between microbes. Overall, the MicroNet-MIMRF method was highly effective at inferring microbial networks, successfully overcoming the challenge of zero-inflation and accurately estimating both linear and non-linear associations between microbial taxa. A comparative analysis with PCC, SCC, SparCC, MENA, and HARMOMIES highlighted the superior performance of MicroNet-MIMRF in terms of AUC and MCC, particularly in scenarios involving complex network structures and zero-inflated data.

### 3.2 Case study

In recent years, the growing prevalence of inflammatory bowel disease (IBD) has imposed significant social and economic burdens on numerous countries, exacerbating the challenges faced by health systems (GBD 2017 Inflammatory Bowel Disease Collaborators 2020). IBD is a non-communicable chronic gastrointestinal disease that encompasses two primary forms: Crohn’s disease and ulcerative colitis. IBD also serves as a risk factor for colorectal cancer (Olén *et al.* 2020, Xi and Xu 2021), a disease closely associated with the gut microbiota. Gut microbiota play a key role in maintaining human health; an imbalance in these microbiota, known as dysbiosis, can trigger the onset of IBD (Glassner *et al.* 2020, Lavelle and Sokol 2020). In this case study, we analyzed the IBD dataset available from the Inflammatory Bowel Disease Multi'omics Database (http://ibdmdb.org). Microbial abundance data were extracted from 399 patients with IBD and 230 healthy controls. The patient group included 75 microbial genera, whereas the healthy group included 53 genera.

**Figure 4.**
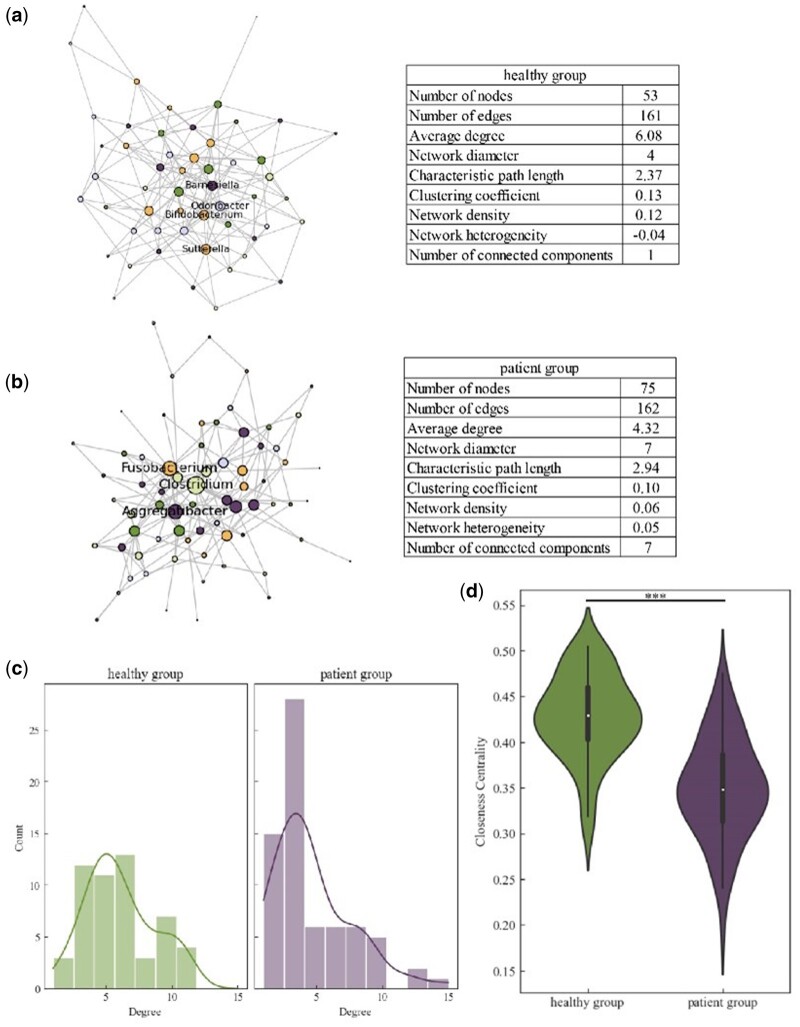
Case study of inflammatory bowel disease (IBD) data. (a, b) Inferred microbial networks for healthy individuals and patients with IBD, respectively. (c) Node degree distribution of microbial networks. (d) Violin plot of closeness centrality of microbial networks. Detailed information on both types of networks is provided in Supplementary Tables S1 and S2 in the supplementary materials.

We applied the MicroNet-MIMRF framework to the dataset to infer the microbial networks. The estimated networks for the healthy and IBD groups are shown in Fig. 4a and b. To confirm the robustness of MicroNet-MIMRF, we split healthy data into two datasets, as well as IBD data, no significant differences were observed between the degree distributions of the two datasets (KS* *=* *1.18, 1.58, all *P *>* *.05). These results indicated the microbial networks constructed by MicroNet-MIMRF were robust. Each node corresponds to a genus, and the node size reflects the degree of the node in the network. In microbial networks, the degree of the node indicates the frequency of interactions or associations between microbes. The average degree of the healthy and IBD microbial networks was 6.08 and 4.32, respectively. This suggests that the average level of interaction among microbes varied between the two networks. Figure 4c displays the node degree distribution in these microbial networks. The Kolmogorov–Smirnov test indicated significant differences between the two distributions (KS* *=* *0.34, *P *<* *.05). The closeness centrality reflects the average shortest path length from one node to other nodes in the network, as shown in Fig. 4d. The Mann-Whitney *U* test revealed a statistically significant difference in closeness centrality between the IBD and healthy groups (*U *=* *3139.0, *P *<* *.001). The closeness centrality of the healthy group was higher than that of the IBD group, implying that the efficiency of information transmission within the gut microbiome of healthy individuals was higher than that in individuals with IBD.

In the microbial networks we constructed, we observed that *Clostridiales noname* had a high degree of healthy network. However, in the IBD network, it was an isolated node with a degree of 0. *Clostridium difficile*, a member of the *Clostridiales* order, is a common cause of diarrhea. The incidence of *C.difficile* infections has been proven to be associated with IBD (Balram *et al.* 2019). *C.noname* is an unknown genus within *Clostridiales* order. In our study, we identified a significant difference in the degree of *C.noname* between healthy and IBD microbial networks. In the healthy network, we discovered a novel association: *C.noname* was associated with *Coprobacillus*. *Coprobacillus*, a part of gut microbiota and belonging to *Clostridiales* order, is known to produce butyric acid (Zhao *et al.* 2021), and may play an important role in maintaining intestinal health (Shi *et al.* 2018). It has been reported as a potential biomarker for distinguishing between colorectal cancer patients and healthy individuals (Yang *et al.* 2020). However, the association between *C.noname* and *Coprobacillus* is rarely reported. In the IBD network, *C.noname* served as an isolated node, with no significant associations detected. This distinction between the two networks suggests the importance of *C.noname*’s potential interactions with other microbes in mediating the development of IBD.

Thus, these findings suggest significant differences in the gut microbiota of healthy individuals and those with IBD, both in terms of function and structure. Our results align with those of previous reports (Alam *et al.* 2020, Chen *et al.* 2020) showing that the gut microbiota undergoes significant alterations in patients with IBD. This demonstrates the robustness and practicality of the MicroNet-MIMRF framework for inferring microbial networks. Moreover, this case study provides valuable insights into gut microbiota alterations in patients with IBD. Nevertheless, these findings require additional biological and experimental validation to clarify their implications.

## 4 Discussion

Advances in high-throughput sequencing technology have significantly enhanced our ability to collect vast amounts of microbiome data. This progress has enabled the study of interactions and associations within microbial communities in various environments, providing valuable and significant information for subsequent laboratory research. Network analysis has become a powerful tool for estimating the associations and interactions among microbes from microbial abundance data. However, microbial abundance data are characterized by zero-inflation and contain many non-linear associations; these two issues hinder the accuracy of predicted microbial networks. Microbial abundance data are typically sparse (Zhang and Yi 2020), which leads to excess extra zeros in the data owing to insufficient sequencing depth and shallow coverage. This is commonly known as the zero-inflation phenomenon in microbial abundance data (Campbell 2021). Directly estimating the relationships between microbes based on traditional correlation methods may result in false associations and overestimation owing to the zero-inflation phenomenon (Ling *et al.* 2021, Ullmann *et al.* 2023). Thus, addressing the issue of zero-inflation is a prerequisite for robust microbial network inference. Although network analysis is a powerful tool for uncovering associations within microbial communities, its biological interpretation and significance have been challenged (Faust 2021). One main reason is that these relationships are too complex to be simply represented. The links in microbial networks are both linear and non-linear, implying interactions such as cross-feeding, quorum sensing, cell signal transduction, and biofilm formation. Traditional correlation inference methods mainly capture linear correlations; however, they failed to estimate non-linear associations. Some complicated interactions, such as biodiversity-nutrient cycling, must be characterized jointly through linear and non-linear relationships (Mo *et al.* 2022). Therefore, estimating the non-linear relationships in microbial networks is crucial for understanding their structure and function.

In this study, we introduce a novel microbial network inference approach named MicroNet-MIMRF. To accurately estimate pairwise associations from the microbial abundance data, we employed ZIP distribution to model the original microbial abundance data. After transforming the abundance count data into discrete data, we avoided the bias resulting from excessive extra zeros. The overperformance of AUC and MCC values in the simulated experiments illustrates that the MicroNet-MIMRF effectively addresses the prevalent issue of zero-inflation in microbial count data. An MRF based on mutual information enabled the MicroNet-MIMRF to estimate the conditional dependencies among microbial taxa, filter significant associations, and recover the network structure. The different network structures suggest that MicroNet-MIMRF captures both linear and non-linear associations among microbes. Furthermore, MicroNet-MIMRF is applicable to a wide range of sample sizes scale; specifically, it can be applied to microbial abundance data of small sample sizes. The efficacy of the MicroNet-MIMRF was validated through experiments using both simulated and actual datasets. Compared with other common methods, MicroNet-MIMRF outperforms under both high ZIRs and complex network structures. Although these differences were observed in other research (Friedman and Alm 2012), no differences were observed in AUC and MCC between PCC, SCC, and SparCC in our results. We considered these differences absent due to the failure in estimating accurate associations for high zero-inflated data of PCC, SCC, and SparCC.

However, similar to most microbial network inference techniques, the MicroNet-MIMRF generates a static microbial network. Although incorporating temporal factors to construct a dynamic microbial network can provide valuable insights into the evolution of microbial communities, our approach does not support the creation of such networks. Therefore, future extensions of MicroNet-MIMRF should include temporal dimensions to enable the construction of dynamic microbial networks. Another limitation of our method is that it relies solely on microbiome data for network construction. For instance, 16 s rRNA sequencing provides information about the presence and abundance of microbes but does not give insight into their functional capabilities. We can only infer indirect interactions or associations among microbes; however, we cannot infer direct interactions (e.g. cooperation, competition, predation) using these data. Merely using microbial abundance data is insufficient for constructing directed networks, which are necessary to reveal more detailed information about microbial interactions. Therefore, future research should focus on enhancing our methodology by incorporating multi-omics data such as metagenomics, transcriptomics, and metabolomics. This integration would allow the construction of more comprehensive and accurate microbial networks. By addressing these limitations, we can improve the robustness and applicability of microbial ecology research, as well as its implications for various environments and health conditions.

## 5 Conclusion

We proposed a novel approach called MicroNet-MIMRF for inferring microbial networks based on mutual information and MRFs. This method can effectively construct a robust microbial network from abundance data, while handling zero-inflation and capturing non-linear correlations. Moreover, we demonstrate its ability to recover the network structures of microbial communities based on microbiome abundance data. This study advances our understanding of microbial interactions and associations and provides novel insights into microbiome research.

## Supplementary Material

vbae167_Supplementary_Data

## Data Availability

The data underlying this article are available at https://github.com/Fionabiostats/MicroNet-MIMRF.
