# Supplementary figures and images for: MicroNet-MIMRF: a microbial network inference approach based on mutual information and Markov random fields
Source: Bioinform Adv. 2024 Oct 28;4(1):vbae167. doi: 10.1093/bioadv/vbae167 (PMC11549015; doi:10.1093/bioadv/vbae167)

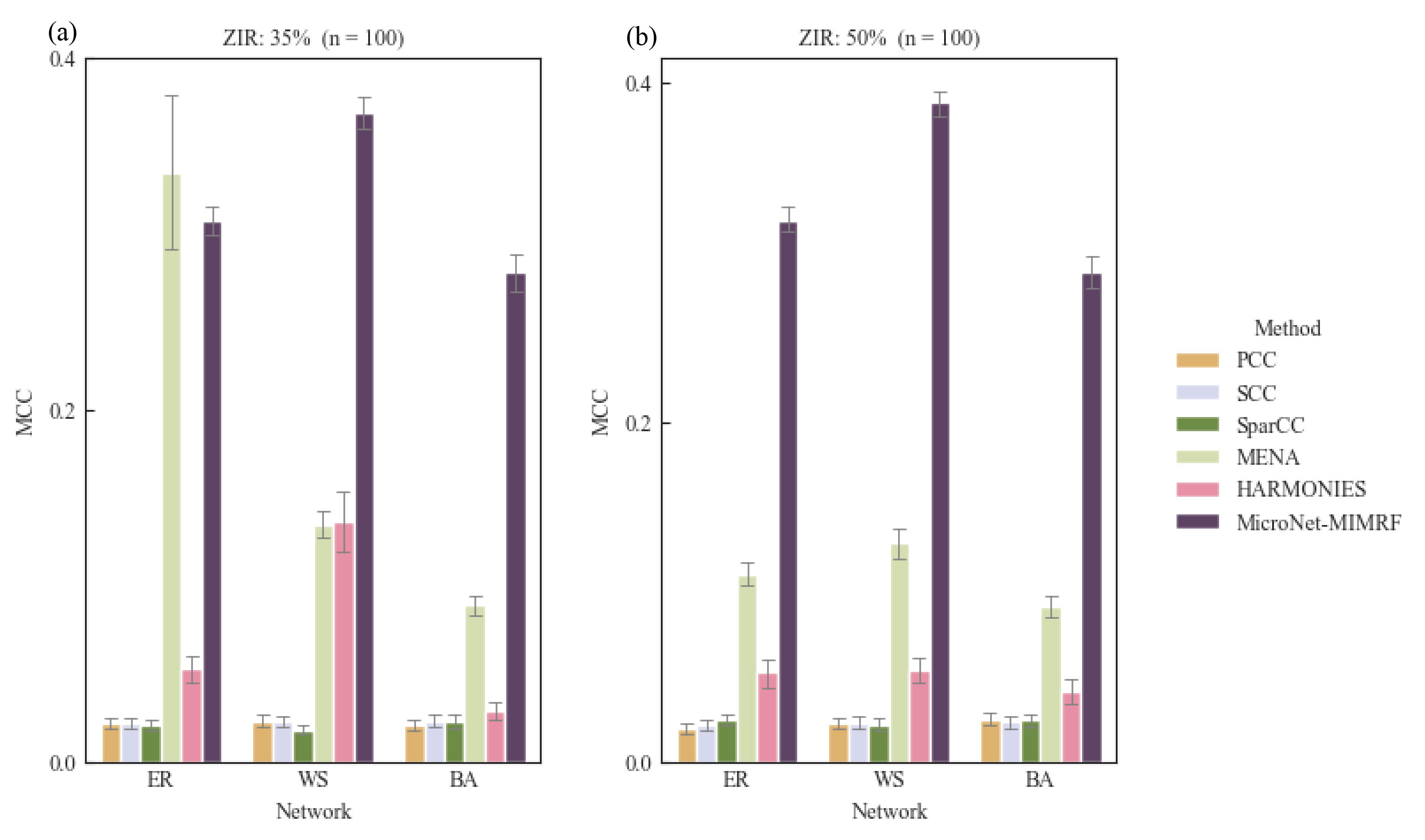

Supplement: vbae167_Supplementary_Data [file vbae167_supplementary_data.zip › Figure_S4.jpg]

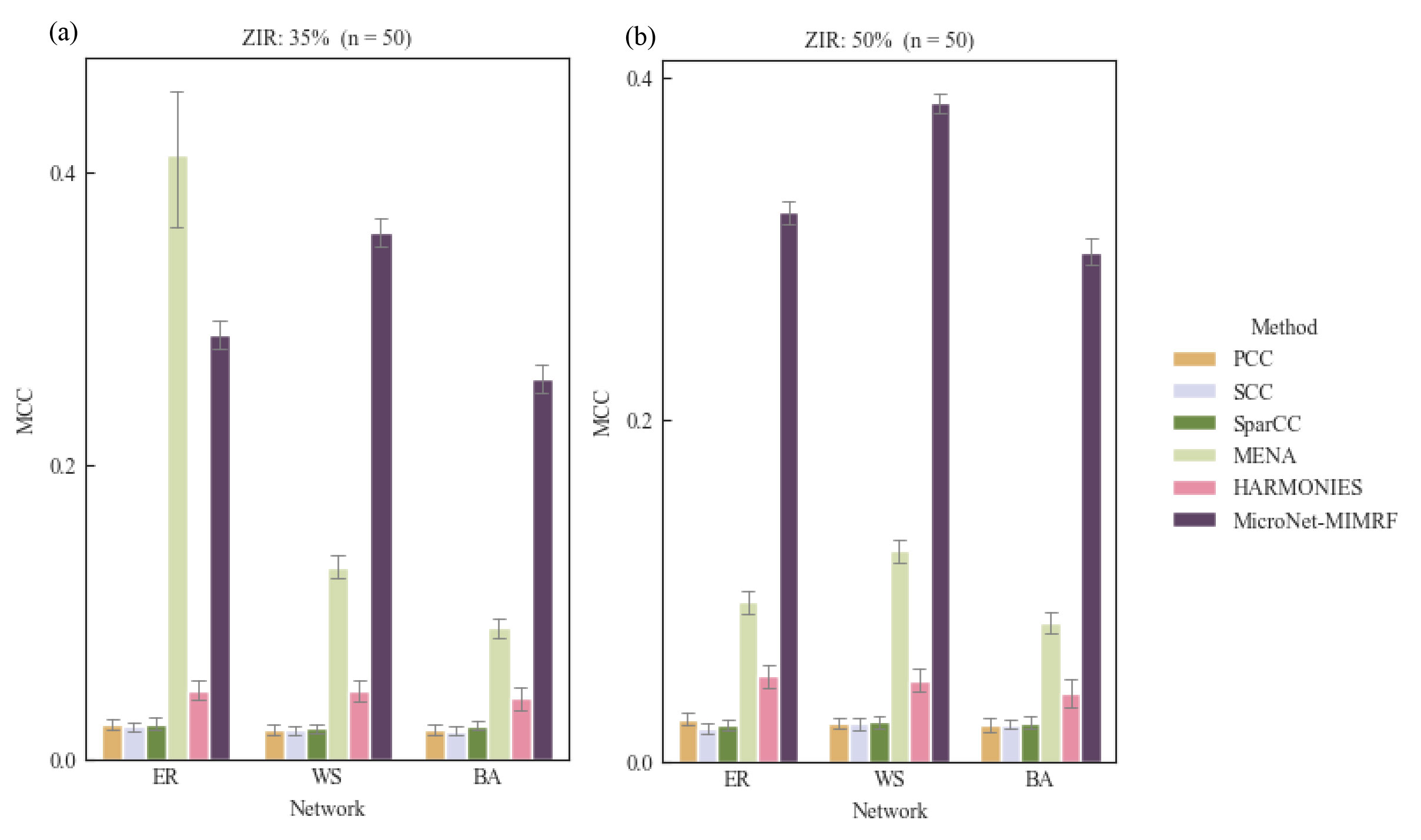

Supplement: vbae167_Supplementary_Data [file vbae167_supplementary_data.zip › Figure_S3.jpg]

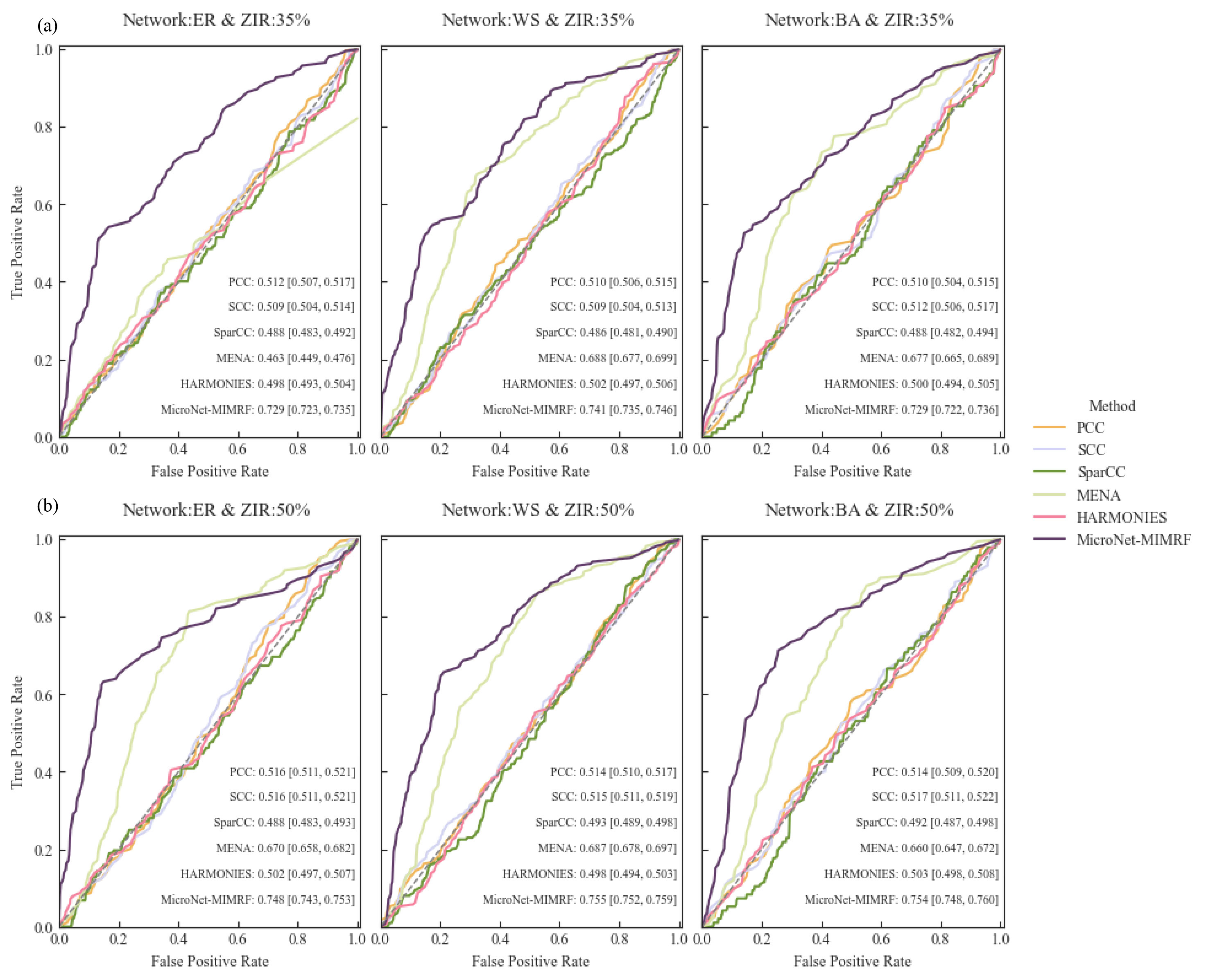

Supplement: vbae167_Supplementary_Data [file vbae167_supplementary_data.zip › Figure_S1.jpg]

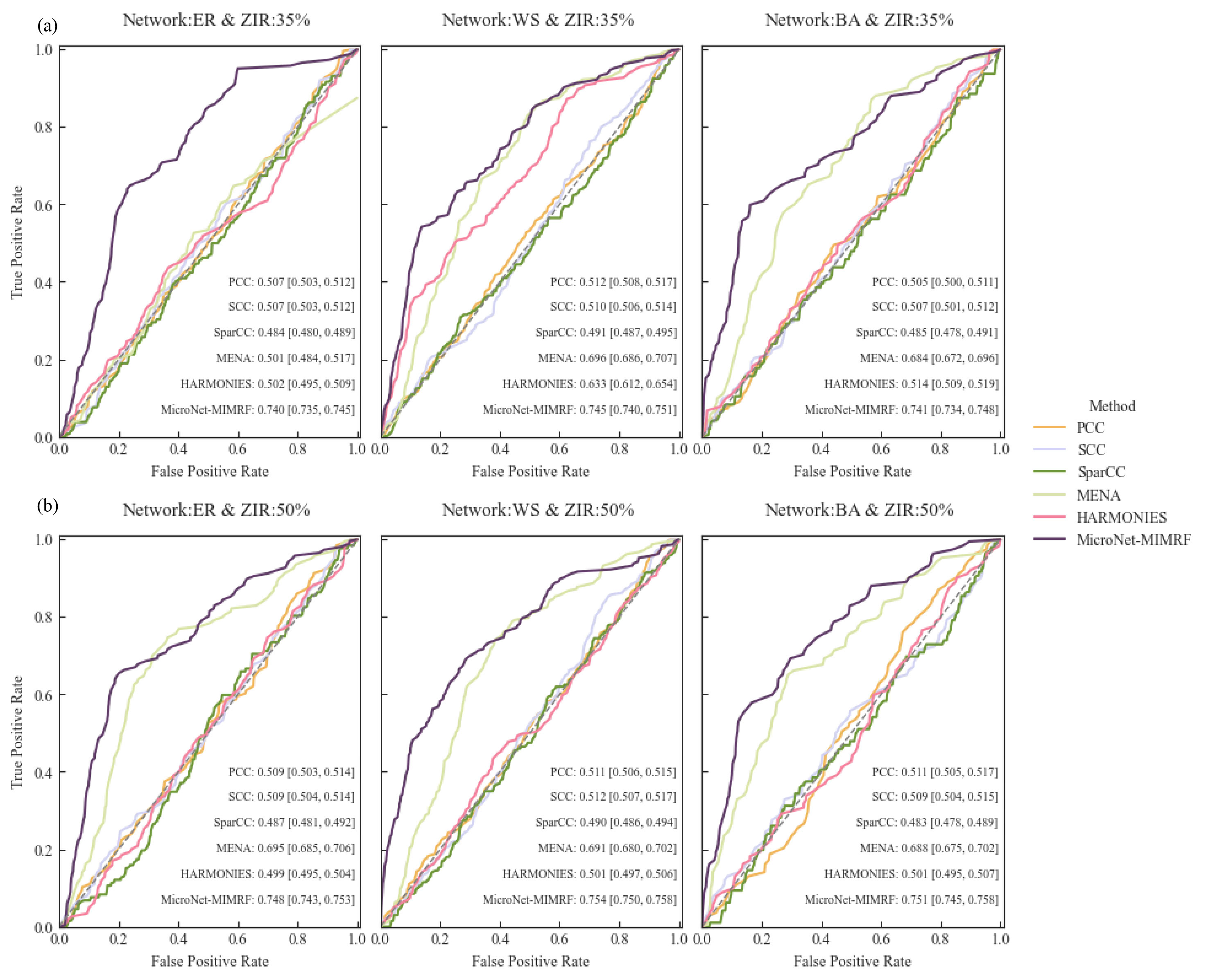

Supplement: vbae167_Supplementary_Data [file vbae167_supplementary_data.zip › Figure_S2.jpg]
